# PABPC4 Broadly Inhibits Coronavirus Replication by Degrading Nucleocapsid Protein through Selective Autophagy

**DOI:** 10.1128/Spectrum.00908-21

**Published:** 2021-10-06

**Authors:** Yajuan Jiao, Ning Kong, Hua Wang, Dage Sun, Sujie Dong, Xiaoyong Chen, Hao Zheng, Wu Tong, Hai Yu, Lingxue Yu, Yaowei Huang, Huan Wang, Baokun Sui, Ling Zhao, Ying Liao, Wen Zhang, Guangzhi Tong, Tongling Shan

**Affiliations:** a Shanghai Veterinary Research Institute, Chinese Academy of Agricultural Sciences, Shanghai, People’s Republic of China; b Jiangsu Co-Innovation Center for the Prevention and Control of Important Animal Infectious Disease and Zoonoses, Yangzhou University, Yangzhou, People’s Republic of China; c College of Animal Sciences, Zhejiang University, Hangzhou, People’s Republic of China; d College of Veterinary Medicine, Huazhong Agricultural University, Wuhan, People’s Republic of China; e School of Medicine, Jiangsu University, Zhenjiang, People’s Republic of China; University of Arizona

**Keywords:** N protein, PABPC4, SP1, coronaviruses, selective autophagy

## Abstract

Emerging coronaviruses (CoVs) can cause severe diseases in humans and animals, and, as of yet, none of the currently available broad-spectrum drugs or vaccines can effectively control these diseases. Host antiviral proteins play an important role in inhibiting viral proliferation. One of the isoforms of cytoplasmic poly(A)-binding protein (PABP), PABPC4, is an RNA-processing protein, which plays an important role in promoting gene expression by enhancing translation and mRNA stability. However, its function in viruses remains poorly understood. Here, we report that the host protein, PABPC4, could be regulated by transcription factor SP1 and broadly inhibits the replication of CoVs, covering four genera (*Alphacoronavirus*, *Betacoronavirus*, *Gammacoronavirus*, and *Deltacoronavirus*) of the *Coronaviridae* family by targeting the nucleocapsid (N) protein through the autophagosomes for degradation. PABPC4 recruited the E3 ubiquitin ligase MARCH8/MARCHF8 to the N protein for ubiquitination. Ubiquitinated N protein was recognized by the cargo receptor NDP52/CALCOCO2, which delivered it to the autolysosomes for degradation, resulting in impaired viral proliferation. In addition to regulating gene expression, these data demonstrate a novel antiviral function of PABPC4, which broadly suppresses CoVs by degrading the N protein via the selective autophagy pathway. This study will shed light on the development of broad anticoronaviral therapies.

**IMPORTANCE** Emerging coronaviruses (CoVs) can cause severe diseases in humans and animals, but none of the currently available drugs or vaccines can effectively control these diseases. During viral infection, the host will activate the interferon (IFN) signaling pathways and host restriction factors in maintaining the innate antiviral responses and suppressing viral replication. This study demonstrated that the host protein, PABPC4, interacts with the nucleocapsid (N) proteins from eight CoVs covering four genera (*Alphacoronavirus*, *Betacoronavirus*, *Gammacoronavirus*, and *Deltacoronavirus*) of the *Coronaviridae* family. PABPC4 could be regulated by SP1 and broadly inhibits the replication of CoVs by targeting the nucleocapsid (N) protein through the autophagosomes for degradation. This study significantly increases our understanding of the novel host restriction factor PABPC4 against CoV replication and will help develop novel antiviral strategies.

## INTRODUCTION

Coronaviruses (CoVs), enveloped single-stranded positive-sense RNA viruses, are the largest group of viruses belonging to the *Nidovirales* order (1). The CoVs are divided into four groups (alpha, beta, gamma, and deltacoronaviruse) by phylogenetic clustering (2). They can infect various hosts, including humans and animals (dogs, cats, chickens, cows, and pigs), causing enteric, respiratory, neurological, and hepatic diseases. The human coronaviruses (HCoVs) were thought to cause mild, self-limiting respiratory infections before the severe acute respiratory syndrome coronavirus (SARS-CoV) outbreak (3). The SARS outbreak in China in 2002 and the Middle East Respiratory Syndrome-CoV emerging in the Middle East in 2012 as a worldwide epidemic virus caused a series of highly pathogenic respiratory syndromes with severe morbidity and high mortality rates (4–7). The outbreak of emerging SARS-CoV-2 disease (COVID-19) has recently been brought to global attention and declared a pandemic by the World Health Organization on 11 March 2020 (4, 8, 9). The avian coronavirus, infectious bronchitis virus (IBV), caused poor weight gain and reduced egg production in chickens (10). The swine coronavirus, porcine epidemic diarrhea virus (PEDV), transmissible gastroenteritis virus (TGEV), porcine deltacoronavirus (PDCoV), and swine acute diarrhea syndrome coronavirus (SADS-CoV) cause severe gastroenteritis in neonatal pigs leading to significant morbidity and mortality (11–15). Coronaviruses have the potential to spread rapidly and cause significant disease in humans and animals, but there is no widely available antiviral drug or vaccine that could be used for the treatment or prevention of the resulting infections.

The CoV genome typically encodes two polyproteins (pp1a and pp1ab) and four main structural proteins (spike protein, S; envelope protein, E; membrane protein, M; and nucleocapsid protein, N) (1). The S protein is a class I fusion protein responsible for virus attachment and membrane fusion (16). The N protein is abundantly expressed within infected cells; plays an important role in virus replication and immune evasions (17, 18), such as binding to the viral RNA to form a helical nucleocapsid; and is involved in transcription, translation, and viral assembly (19–22). Therefore, the N protein may serve as a useful drug target for treating viral infections (23, 24).

Macroautophagy/autophagy is a major degradative process for maintaining cellular homeostasis by degrading the aggregated proteins or organelles and misfolded or long-lived cytoplasmic proteins during periods of starvation in eukaryotic cells (25). During autophagy, the misfolded proteins damaged organelles or invading pathogens, as substrate proteins are modified with ubiquitin and recognized by cargo receptors (25, 26). The cargo receptors, including SQSTM1/p62, NDP52/CALCOCO2 (nuclear dot protein 52 kDa), OPTN (optineurin), and NBR1 (neighbor of BRCA1), recognize the ubiquitin-binding substrate proteins and directly interact with ATG8 (autophagy-related protein 8) for selective packaging of their targets into the autophagosome. Then, the autophagosomes fuse with lysosomes to degrade the cargo and release metabolic by-products, such as amino acids (27). In addition to its physiological functions in cellular homeostasis, autophagy has been shown to play an important role in innate immunity and viral replication (28, 29). For example, autophagy inhibits herpes simplex virus 1 (HSV-1) replication by protein kinase R (PKR)-dependent autophagic degradation (30). However, foot-and-mouth disease virus (FMDV)-induced autophagy facilitates viral replication through its capsid protein VP2, which activates the EIF2S1-ATF4 pathway to decrease the aggregation of HTT103Q (huntingtin polyglutamine expansion protein) (31, 32). CoVs induce autophagy in viral replication (33–35), but the underlying mechanism of the cross talk between autophagy and CoV replication is yet to be understood.

Poly(A)-binding protein (PABP), a nucleocytoplasmic shuttling protein, has a fundamental role in promoting gene expression by enhancing mRNA translation and stability (36, 37). Poly(A)-binding protein bound to the 3′ end of the mRNA via the polyadenylated [poly(A)] tail and 5′ cap structure of the mRNA through interactions with eukaryotic initiation factor 4G (eIF4G) to form a “closed loop” (38–40). This special PABP and mRNA complex could enhance ribosome subunit recruitment to promote translation initiation and protect mRNAs from exonucleolytic degradation (41, 42). Poly(A)-binding protein, predominantly present in the cytoplasm, preferentially accumulates in the nucleus during heat shock and oxidative stress (43, 44). PABP could be driven into the nucleus upon viral infections from viruses such as HSV-1 and rotavirus (45, 46). Several studies have investigated that the coronavirus N proteins bind to PABP, and PABP binding by the N protein of bovine coronavirus (BCoV) negatively regulates translation of coronaviral RNA and host mRNA both *in vitro* and in cells (47–49). Poly(A)-binding protein 4 (PABPC4), one of the homologs of PABP, was initially identified as a human T-cell activation-induced protein (50), but its role in cellular processes and virus infection has not been further explored. In this study, we demonstrated that the expression of PABPC4 was regulated by transcription factor SP1 during PEDV infection. In addition, PABPC4 inhibited CoV replication by targeting and degrading the CoV N protein through selective autophagy. Furthermore, we revealed that PABPC4 utilized E3 ubiquitin ligase MARCH8 to ubiquitinate CoV N protein, which was delivered by cargo receptor NDP52 to autophagosomes for selective degradation. Our findings revealed a new antiviral mechanism for PABPC4, by which it suppresses CoV replication through selective autophagy.

## RESULTS

### PEDV infection downregulates the expression of PABPC4 via the transcription factor of SP1.

**FIG 1:**
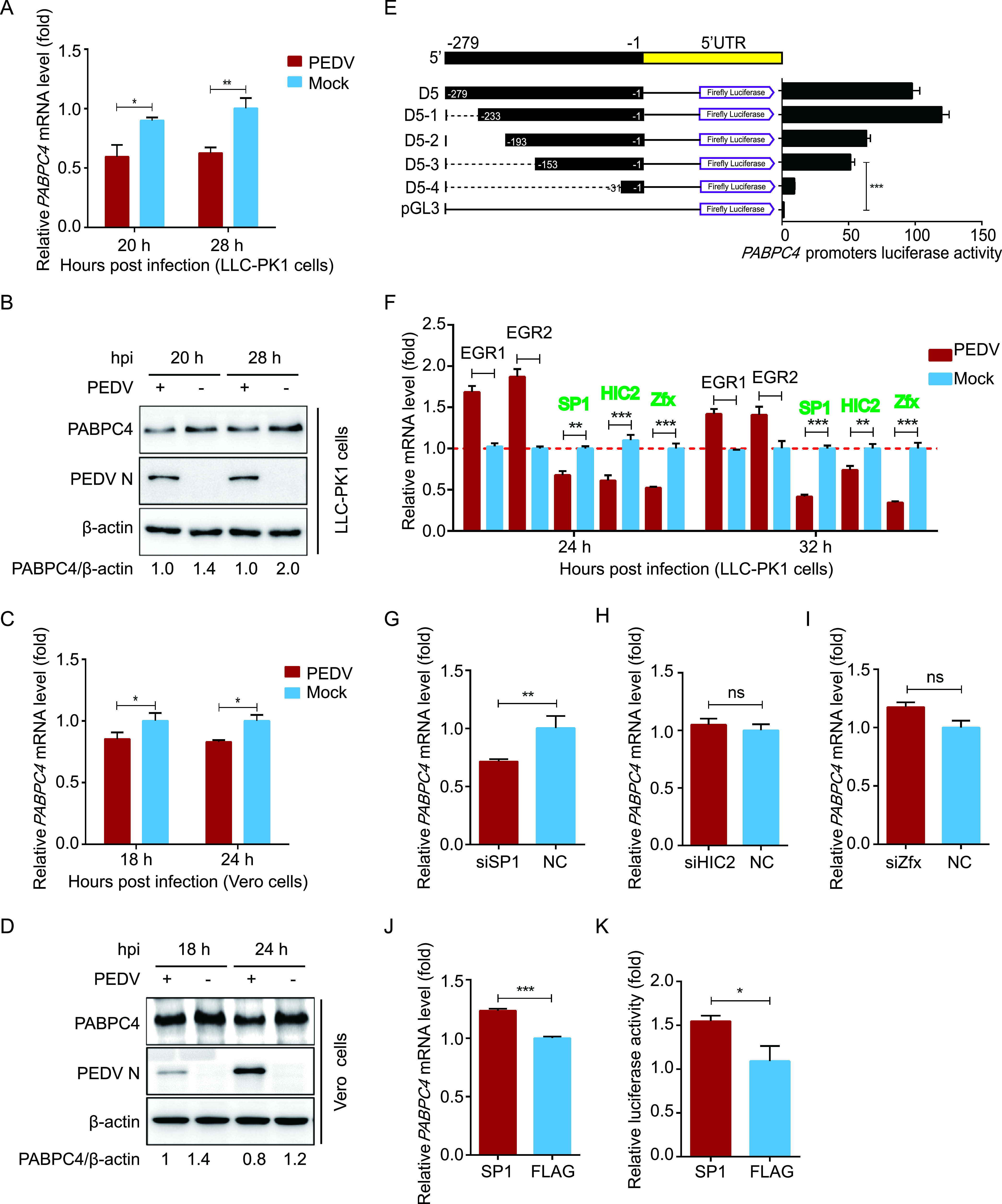
PEDV infection downregulates the expression of PABPC4 by the transcription factor of SP1. (A and B) LLC-PK1 cells were infected with PEDV at an MOI of 1 and harvested at indicated times. The mRNA level and protein level of PABPC4 were analyzed by real-time PCR and Western blotting. (C and D) Vero cells were infected with PEDV at an MOI of 1 and harvested at indicated times. The mRNA level and protein level of PABPC4 were analyzed by real-time PCR and Western blotting. (E) Series of truncated *PABPC4* promoter constructs (D5-1 to D5-4) and *Renilla* luciferase reporter vector (pRL-TK-Luc) were transfected into 293T cells and analyzed for dual luciferase activity. (F) *EGR1*, *EGR2*, *SP1*, *HIC2*, and *Zfx* mRNA levels in the same samples (A) were analyzed by real-time PCR. (G, H, I, and J) Vero cells were transfected with *SP1* siRNA, *HIC2* siRNA, *Zfx* siRNA, or the plasmid encoding FLAG-SP1. Twenty-four hours posttransfection, the transcription levels of *PABPC4* were analyzed with real-time PCR. (K) 293T cells were transfected with FLAG-SP1 and *PABPC4* promoter plasmids and a *Renilla* Luciferase reporter vector. The cells were analyzed for dual luciferase activity. Data are represented as means ± SD of triplicate samples. *, *P *< 0.05; **, *P *< 0.01; ***, *P *< 0.001 (two-tailed Student’s *t* test).

To screen the potential proteins for their ability to inhibit PEDV replication, we used the proteomics approach of isobaric tagging for relative and absolute quantitation (iTRAQ) to analyze the proteins in PEDV-infected cells and uninfected cells as previously described (51). The results showed that PABPC4 was downregulated in Vero cells during PEDV infection. Porcine kidney cells (LLC-PK1) and Vero cells were infected with PEDV (strain JS-2013) at an multiplicity of infection (MOI) of 1, and the cell lysates were analyzed using quantitative real-time PCR (qRT-PCR) to confirm the expression of PABPC4. As shown in Fig. 1A and C, the expression of PABPC4 mRNA was downregulated in both LLC-PK1 and Vero cells in response to PEDV infection, relative to that in mock-infected cells. We also showed that the PABPC4 protein level was significantly decreased in PEDV-infected cells (Fig. 1B and D). Thus, PEDV infection reduces the expression of PABPC4.

The accumulation of PABPC4 in the cytoplasm may be affected by heat shock, HSV-1, and murine gammaherpesvirus 68 (MHV68) (43, 46, and 52), but the mechanisms by which transcription factors regulate the expression of PABPC4 are not fully understood. To identify the transcription factors of PABPC4, we amplified 1,350 bp of the *PABPC4* promoter and cloned it into a luciferase vector (pGL3-Basic). Truncated promoters (designated D1 to D5) were cloned, and their ability to induce luciferase expression in HEK 293T cells was tested. A promoter fragment containing the nucleotides −279 to −1 (D5) induced luciferase activity comparable to that of the full-length promoter. In contrast, constructs that lacked the sequence −279 to −1 induced low or undetectable luciferase expression. These results suggested that the *PABPC4* core promoter region lies between positions −279 and −1. To further confirm the boundaries of the minimal *PABPC4* promoter, a series of truncated promoters (designated D5-1 to D5-4), based on the sequence from −279 to −1, were cloned into the luciferase vector. The construct containing nucleotides from −153 to −1 (D5-3) showed increased luciferase expression (Fig. 1E). Altogether, these results indicate that the minimal *PABPC4* core promoter is located between −153 and −1.

To analyze the transcriptional regulation of *PABPC4*, we examined the promoter for potential transcription factor-binding sites (TFBSs) using the JASPAR vertebrate database (http://jaspar.genereg.net/) (34). We found that the minimal PABPC4 core promoter region (approximately −153 to −1 bp) contains several TFBSs, including Zfx-, HIC2-, EGR1-, EGR2-, and SP1-binding sites. Next, we detected the mRNA levels of all of the putative transcription factors with qRT-PCR and found that only *SP1*, *HIC2*, and *Zfx* were downregulated following PEDV infection (Fig. 1F). To further evaluate whether these transcription factors were involved in the regulation of *PABPC4* expression, we synthesized small interfering RNAs (siRNAs) targeting their mRNA sequences. The qRT-PCR analysis revealed that the level of *PABPC4* mRNA was significantly reduced in Vero cells transfected with *SP1* siRNA (Fig. 1G) but remained stable in cells transfected with *HIC2* or *Zfx* siRNA (Fig. 1H and I). Subsequently, we overexpressed the putative transcription factor SP1 to investigate the inducible expression of endogenous *PABPC4*. qRT-PCR revealed that SP1 overexpression significantly enhanced *PABPC4* transcript levels relative to that observed following control vector transfection (Fig. 1J), and it induced higher luciferase expression with the promoter containing nucleotides from −153 to −1 (D5-3) (Fig. 1K). Overall, these results indicate that SP1 plays an important role in inducing PABPC4 expression by binding to the PABPC4 promoter.

### PABPC4 inhibits PEDV replication.

**FIG 2:**
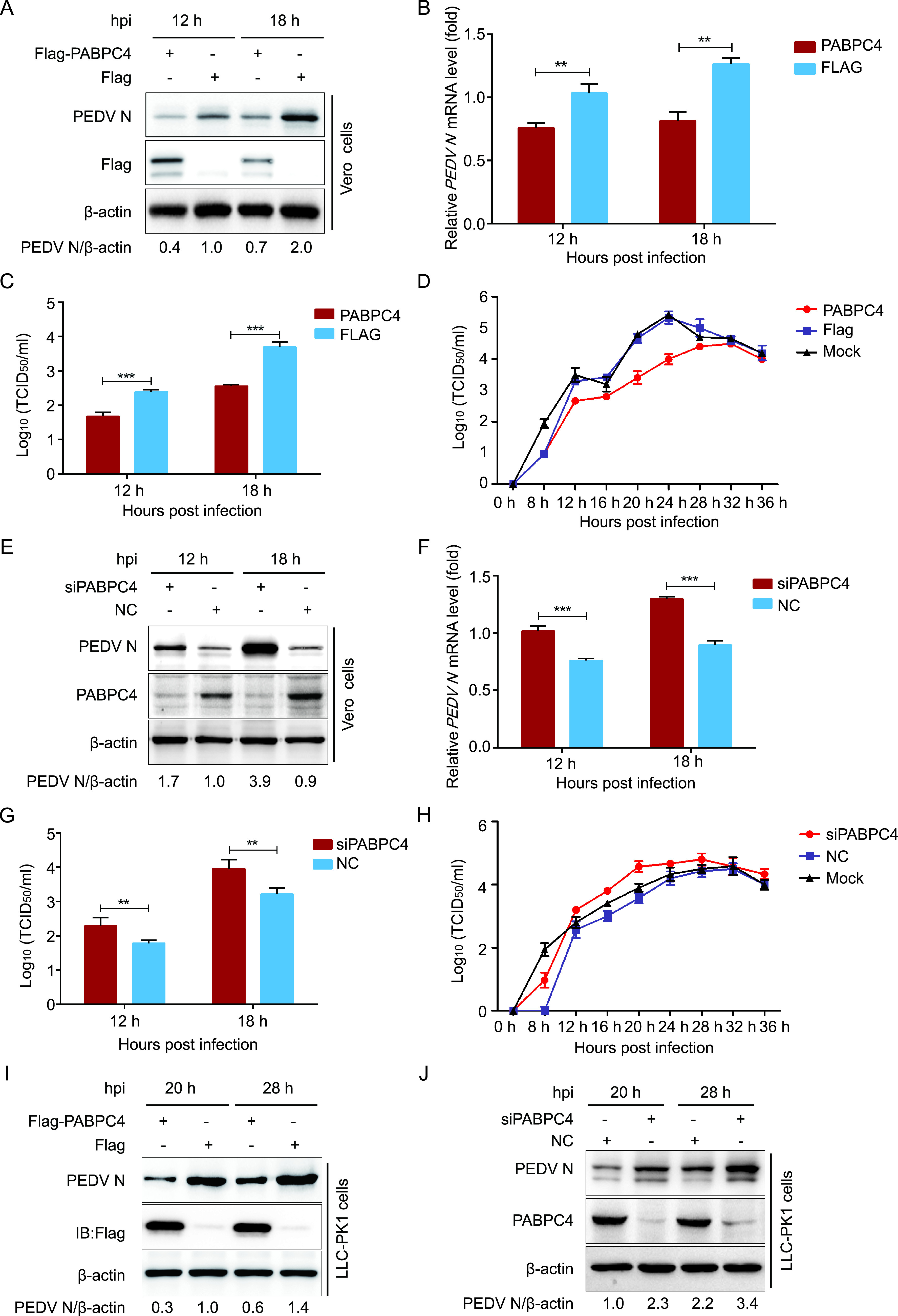
PABPC4 inhibits PEDV replication. (A) Vero cells were transfected with plasmid encoding FLAG-PABPC4 or the FLAG tag. The cells were infected with PEDV at an MOI of 0.01 after 24 h posttransfection and harvested at the indicated times. The N protein was analyzed with Western blotting. β-actin was used as the sample loading control. (B) The PEDV *N* mRNA in the same samples (A) were analyzed by real-time PCR. (C and D) PEDV titers in the culture supernatants of the Vero cells treated as described in panel A were measured as 50% tissue culture infective dose (TCID_50_). (E) *PABPC4* siRNA or negative control siRNA were transfected into Vero cells, and then infected with PEDV at an MOI of 0.01. The N protein was analyzed with Western blotting. β-actin was used as the sample loading control. (F) The PEDV *N* mRNA in the same samples (E) were analyzed by real-time PCR. (G and H) PEDV titers in the culture supernatants of the Vero cells treated as described in panel E were measured as TCID_50_. (I and J) FLAG-PABPC4 vector or *PABPC4* siRNA were transfected into LLC-PK1 cells, and then infected with PEDV at an MOI of 1. The N protein was analyzed with Western blotting. β-actin was used as the sample loading control. Data are represented as means ± SD of triplicate samples. **, *P < *0.01; ***, *P < *0.001 (two-tailed Student’s *t* test).

Vero cells were transfected with PABPC4-FLAG for 24 h and infected with PEDV at an MOI of 0.01 to investigate whether PABPC4 inhibits PEDV replication. The cells and supernatants of the infected cell cultures were collected at various time points and assayed for levels of viral mRNA and N protein as well as viral titers. As shown in Fig. 2A, the levels of PEDV N protein were significantly lower in cells overexpressing PABPC4-FLAG than in cells transfected with the control vector. The mRNA of PEDV N in the cells overexpressing PABPC4 was also significantly lower than that in the cells transfected with an empty vector (Fig. 2B). As expected, the viral titers in the supernatants of the cells overexpressing PABPC4 were lower than those from cells transfected with the control vector (Fig. 2C and D). To further verify these conclusions, siRNAs targeting PABPC4 were synthesized and transfected into Vero cells for 24 h, followed by PEDV infection. In cells transfected with *mPABPC4* siRNA, the viral yield was significantly higher than in non-target-control-siRNA-transfected cells (Fig. 2E to H). Moreover, PEDV proliferation in LLC-PK1 cells was not only inhibited by the overexpression of PABPC4 but also significantly higher than that in non-target-control-siRNA-transfected cells (Fig. 2I and J). These results indicate that PABPC4 plays a role in the inhibition of PEDV replication.

### PABPC4 interacts with PEDV N protein and degrades N protein by autophagy.

**FIG 3:**
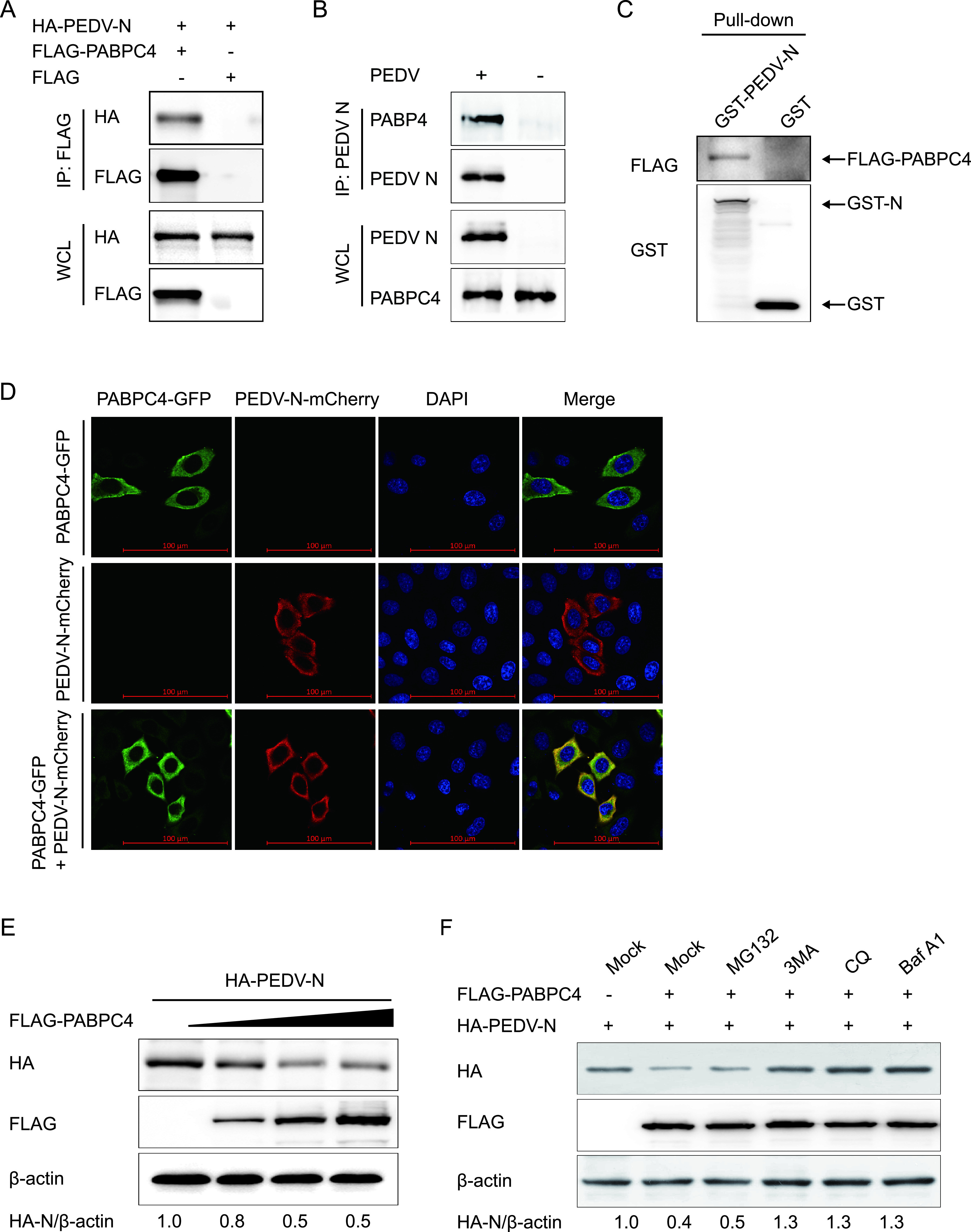
PABPC4 targets and degrades N protein by autophagy. (A) 293T cells were transfected with plasmids encoding FLAG-PABPC4 and HA-N for 24 h, and samples were then harvested and analyzed by co-IP with anti-FLAG binding beads and Western blotting. (B) Vero cells were infected or mock-infected with PEDV at an MOI of 1 and harvested at 24 hours postinfection (hpi). Immunoprecipitation was performed with an anti-PEDV N protein antibody, and Western blotting analysis was performed with a monoclonal antibody against PEDV N protein and an anti-PABPC4 antibody. (C) The PABPC4 protein and N protein were expressed in bacterial strain BL21(DE3) and purified for the GST pulldown analysis. Input, PABPC4. (D) HeLa cells were transfected with plasmids encoding PABPC4-GFP and N-mCherry for 24 h. The cells were fixed and processed for immunofluorescence. Fluorescent signals were observed with confocal immunofluorescence microscopy. Scale bars, 100 μm. (E) The increasing concentrations of a vector expressing FLAG-PABPC4 (wedge) and the vector expressing HA-N were transfected into 293T cells. The cell lysates were analyzed with Western blotting 24 h later. β-actin was used as the sample loading control. (F) Plasmids encoding FLAG-PABPC4 and HA-N were transfected into 293T cells and then treated with MG132 (5 μM), 3MA (0.5 mM), CQ (10 μM), or Baf A1 (0.1 μM) for 8 h. The cell lysates were then analyzed by Western blotting.

To determine the molecular mechanisms by which PABPC4 inhibits PEDV replication, we cotransfected the plasmid encoding FLAG-PABPC4 and PEDV structural proteins (S1, S2, E, M, and N) into HEK 293T cells to identify the PEDV structural protein that interacts directly with PABPC4 by coimmunoprecipitation (co-IP) analysis. The results showed that PABPC4 efficiently coimmunoprecipitated with PEDV N protein (Fig. 3A). Furthermore, PEDV N protein could efficiently coimmunoprecipitate with the endogenous PABPC4 protein (Fig. 3B). A glutathione *S*-transferase (GST) pulldown assay was also used to verify the binding between PABPC4 and PEDV N proteins. The results showed that GST-fused PEDV N (GST-N) could bind to PABPC4, whereas GST alone did not (Fig. 3C), indicating that PEDV N is bound directly to PABPC4. We then investigated the subcellular localization of PABPC4 and the PEDV N proteins with confocal microscopy. Immunofluorescence in HeLa cells transfected with plasmids encoding enhanced green fluorescent protein (EGFP)-PABPC4 (green fluorescence) and mCherry-PEDV-N (red fluorescence) for 24 h revealed that PABPC4 and N proteins colocalized in the cytoplasm (Fig. 3D). These results demonstrated that PABPC4 directly interacts with the PEDV N protein.

To analyze whether PABPC4 is involved in regulating the stability of N proteins, the plasmids encoding hemagglutinin (HA)-N protein and FLAG-PABPC4 were cotransfected into HEK 293T cells, and the N protein abundances were examined by Western blotting. The results showed that the levels of N proteins decreased in a dose-dependent manner in response to PABPC4 (Fig. 3E). These data demonstrated that PABPC4 is involved in regulating PEDV N protein levels.

There are two major intracellular protein degradation pathways in eukaryotic cells: the ubiquitin-proteasomal system and the autolysosome pathway. Plasmids encoding the PEDV N and PABPC4 proteins were cotransfected into HEK 293T cells to assess the degradation pathways of N protein by PABPC4, and the cells were treated with the proteasome inhibitor MG132 and the autophagy inhibitors 3-methyladenine (3MA), chloroquine (CQ), or bafilomycin A_1_ (Baf A1). Western blots showed that PABPC4-mediated degradation of N protein was blocked by the autophagy inhibitors 3MA, CQ, and Baf A1 but not by the proteasome inhibitor MG132 (Fig. 3F). Thus, the degradation of N protein might be mediated by the autophagy-lysosome pathway. These results indicated that PABPC4 interacts with the PEDV N protein and degrades N protein by autophagy.

### PABPC4 degrades PEDV N protein through the PABPC4-MARCH8-NDP52 autophagosome pathway.

**FIG 4:**
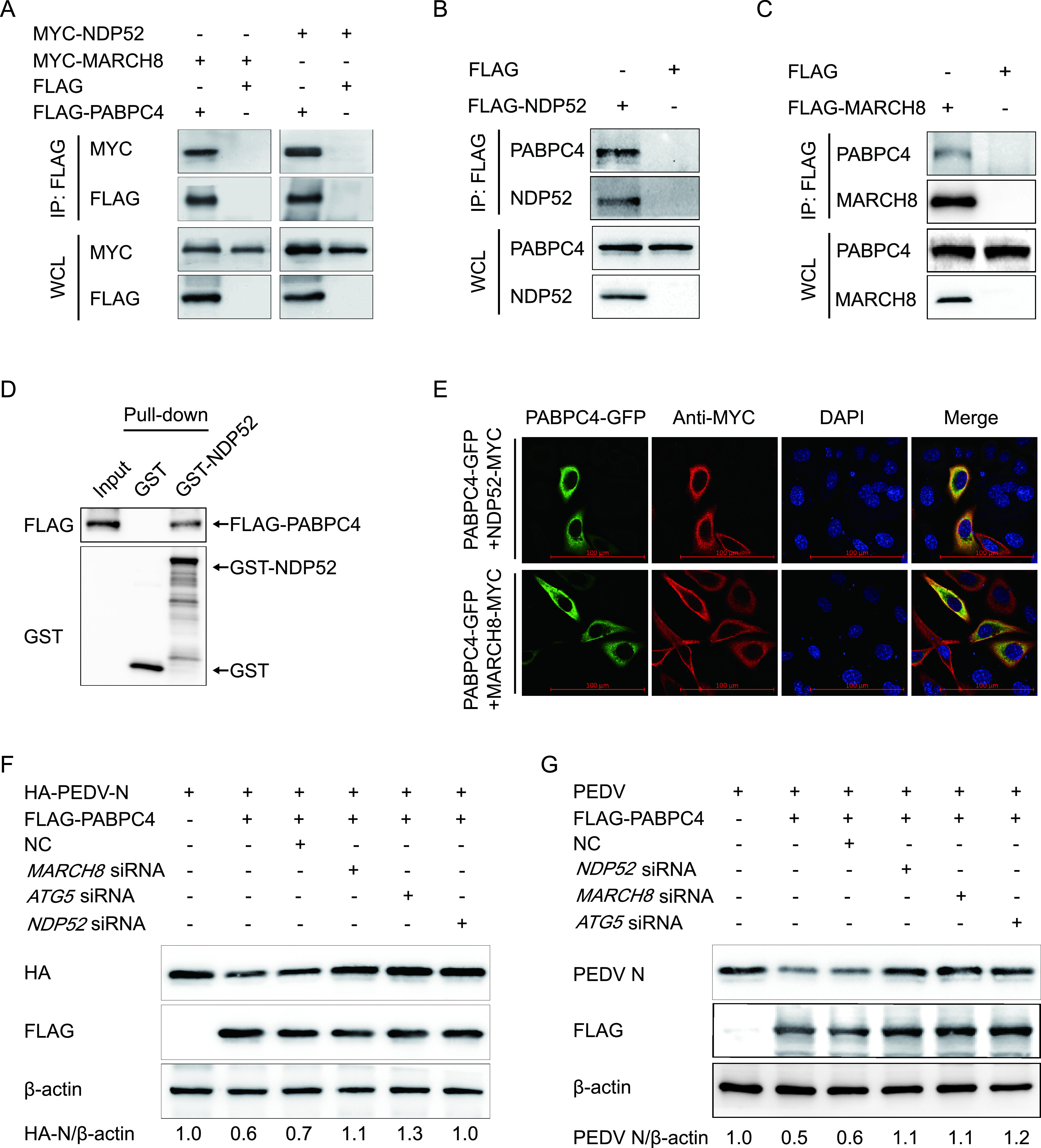
PABPC4 degrades PEDV N protein through selective autophagy. (A) 293T cells were transfected with plasmids encoding FLAG-PABPC4 and MYC-MARCH8 or MYC-NDP52 for 24 h, followed by co-IP with anti-FLAG binding beads and Western blotting with anti-MYC and anti-FLAG antibodies. The whole-cell lysates (WCLs) without immunoprecipitation were analyzed by Western blotting, and β-actin was used as the loading control. (B and C) LLC-PK1 cells were transfected with plasmids encoding FLAG-NDP52 or FLAG-MARCH8 for 24 h, followed by co-IP with anti-FLAG binding beads and Western blotting with anti-PABPC4, anti-NDP52, and anti-MARCH8 antibodies. (D) *FLAG-PABPC4* and *NDP52* genes were cloned into the pCold TF plasmid and pCold GST plasmid, respectively. Recombinant proteins were expressed in bacterial strain BL21(DE3) and purified for the GST pull-down. After adequate washing, proteins eluted from beads were analyzed by Western blotting. Input, FLAG-PABPC4. (E) 293T cells were transfected with plasmids encoding PABPC4-GFP and MYC-MARCH8 or MYC-NDP52 for 24 h and then probed with specific primary and secondary antibodies. Nuclei were stained with DAPI (blue). The fluorescent signals were observed with confocal immunofluorescence microscopy. Scale bars represent 100 μm. (F) 293T cells were cotransfected with small interfering RNA (*NDP52* siRNA, *MARCH8* siRNA, *ATG5* siRNA, or negative-control siRNA) and plasmids encoding FLAG-PABPC4 and HA-PEDV-N and were then analyzed by Western blotting with anti-HA antibody. (G) Vero cells were cotransfected with plasmids encoding FLAG-PABPC4 and small interfering RNA (*NDP52* siRNA, *MARCH8* siRNA, *ATG5* siRNA, or negative-control siRNA) and then infected with PEDV (MOI = 0.01) and harvested at the indicated times. Western blotting was performed with a monoclonal antibody against PEDV N protein.

During selective autophagy, the substrate proteins are first ubiquitinated by an E3 ubiquitin ligase and then recognized by cargo receptors. The cargo receptors deliver the substrates to ATG8 family proteins and form autophagosomes to degrade the substrates. Recent studies have shown that interferon-inducible antiviral factor, BST2, could recruit the E3 ubiquitin ligase MARCH8 to catalyze the ubiquitination of the PEDV N protein, and the cargo receptor NDP52 recognizes and delivers the ubiquitinated N protein to autolysosomes for degradation (34). In this study, we found that PABPC4 degrades the N protein of PEDV via autophagy. Co-IP assays were performed to examine whether PABPC4 mediates the ubiquitination of N protein by recruiting MARCH8 and NDP52. This involved cotransfecting HEK 293T cells with plasmids encoding FLAG-PABPC4 and myc-MARCH8, or myc-NDP52, followed by co-IP using anti-FLAG monoclonal antibody (MAb) affinity gel. Western blotting showed that anti-FLAG MAb affinity gel successfully pulled down FLAG-PABPC4/myc-MARCH8 or FLAG-PABP4/myc-NDP52 (Fig. 4A), demonstrating interactions between FLAG-PABPC4 and myc-MARCH8 as well as FLAG-PABP4 and myc-NDP52. MARCH8 and NDP52 also could coimmunoprecipitate with the endogenous PABPC4 protein (Fig. 4B and C). The GST pulldown further validated the direct binding of PABPC4 to NDP52 (Fig. 4D). We also confirmed the colocalization of PABPC4 with MARCH8 and NDP52 by immunostaining (Fig. 4E). It has been reported that the N protein of PEDV binds to MARCH8 and NDP52 (34). Our data suggested that PABPC4 targets the PEDV N protein and MARCH8 toward autophagic degradation by allowing their binding to the cargo receptor NDP52.

To determine whether MARCH8 and NDP52 are involved in PABPC4-induced autophagic degradation of the N protein, we individually transfected HEK 293T cells with plasmids encoding HA-PEDV-N and FLAG-PABPC4, together with small interfering RNAs (*NDP52*, *MARCH8*, *ATG5*, or negative-control siRNA). At 24 h posttransfection, Western blotting was performed. We found that interfering with the expression of NDP52, MARCH8, or ATG5 effectively prevented the degradation of PABPC4-induced N protein (Fig. 4F). The results also showed that interfering with NDP52, MARCH8, or ATG5 protein expression increased PEDV replication in Vero cells (Fig. 4G). These results suggest that the N protein of PEDV is degraded by selective autophagy through the PABPC4-MARCH8-NDP52 autophagosome pathway.

### PABPC4 inhibits coronavirus replication through degrading N proteins.

**FIG 5:**
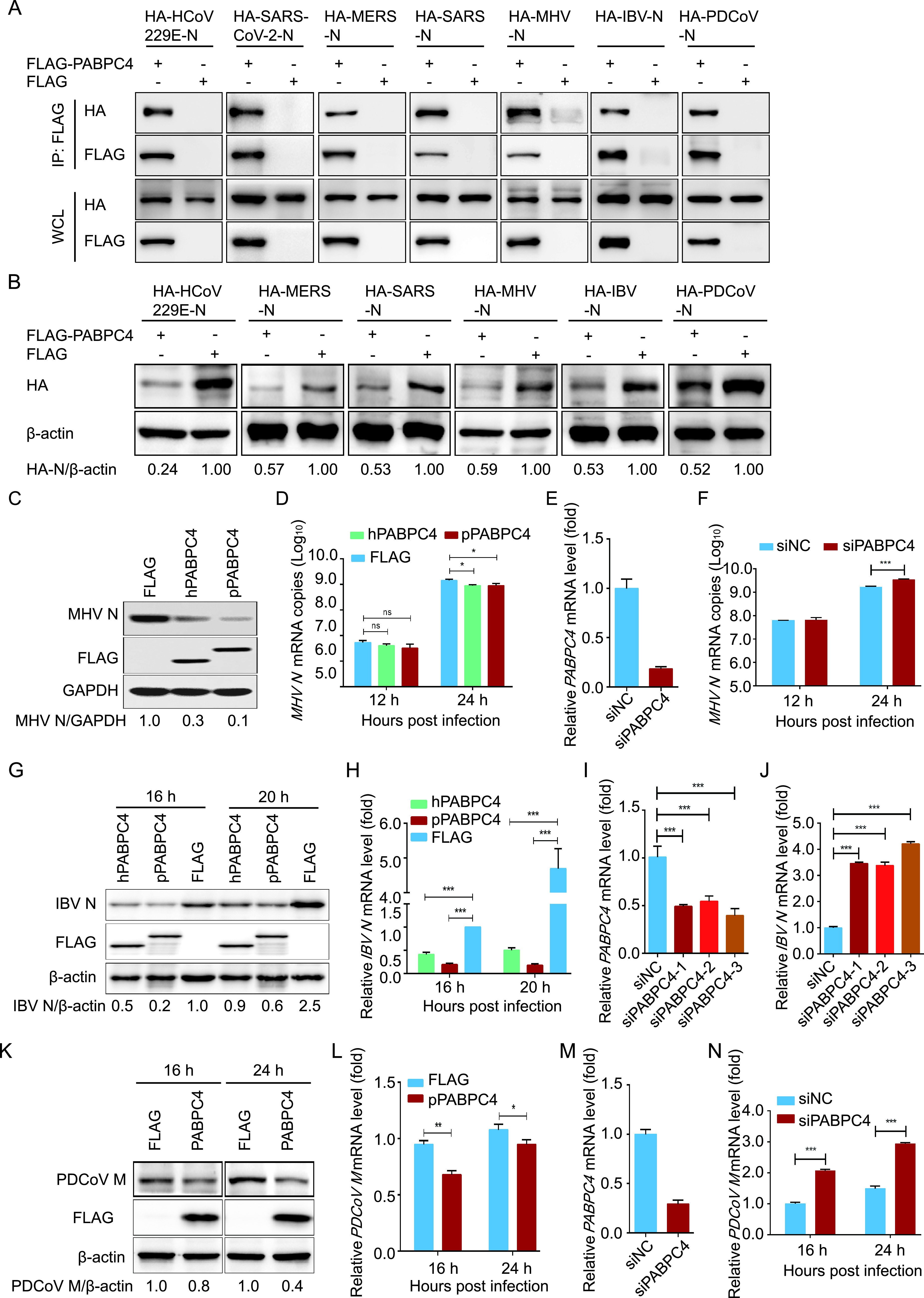
PABPC4 inhibits the coronavirus replication through degraded N proteins. (A) 293T cells were transfected with plasmids encoding coronavirus N proteins (HA-HCov 229E-N, HA-SARS-CoV-2-N, HA-MERS-N, HA-SARS-N, HA-MHV-N, HA-IBV-N, and HA-PDCoV-N) and FLAG-PABPC4 or empty vectors for 24 h, followed by co-IP with anti-FLAG beads and Western blotting with anti-HA and anti-FLAG antibodies. (B) HEK 293T cells were transfected with plasmids encoding coronavirus N proteins (HA-HCov 229E-N, HA-MERS-N, HA-SARS-N, HA-MHV-N, HA-IBV-N, and HA-PDCoV-N) and FLAG-PABPC4 or empty vectors for 24 h. The cell lysates were analyzed by Western blotting with indicated antibodies. (C and D) N2a cells were transfected with plasmids encoding FLAG-human-PABPC4 (FLAG-hPABPC4), FLAG-pig-PABPC4 (FLAG-pPABPC4), or FLAG tag. At 24 h posttransfection, the cells were infected with MHV at an MOI of 0.01 and harvested at the indicated times. The cell lysates were analyzed by Western blotting and real-time PCR, respectively. GAPDH was used as the loading control. (E) The knockdown efficiency of the PABPC4 siRNAs in N2a cells was analyzed by real-time PCR. (F) N2a cells were transfected with PABPC4 siRNA and infected with MHV (MOI = 0.01). MHV replication was evaluated by real-time PCR assay. (G and H) H1299 cells were transfected with plasmids encoding FLAG-hPABPC4, FLAG-pPABPC4, or the FLAG tag. At 24 h posttransfection, the cells were infected with IBV at an MOI of 0.01 and harvested at the indicated times. The cell lysates were analyzed by Western blotting and real-time PCR, respectively. (I) The knockdown efficiency of the PABPC4 siRNAs in H1299 cells was analyzed by real-time PCR. (J) H1299 cells were transfected with PABPC4 siRNA and infected with IBV (MOI = 0.01). IBV replication was evaluated by a real-time PCR assay. (K and L) ST cells were transfected with plasmids encoding FLAG-PABPC4 or the FLAG tag. At 24 h posttransfection, the cells were infected with PDCoV at an MOI of 0.01 and harvested at the indicated times. The cell lysates were analyzed by Western blotting and real-time PCR, respectively. β-actin was used as the loading control. (M) The knockdown efficiency of the PABPC4 siRNAs in ST cells was analyzed by real-time PCR. (N) ST cells were transfected with PABPC4 siRNA and infected with PDCoV (MOI = 0.01). PDCoV replication was evaluated by real-time PCR assay. Data are represented as means ± SD of triplicate samples. *, *P *< 0.05; **, *P *< 0.01; ***, *P *< 0.001 (two-tailed Student’s *t* test).

PEDV belongs to the *Coronaviridae* family, which is divided into the following four major genera: *Alphacoronavirus*, *Betacoronavirus*, *Gammacoronavirus*, and *Deltacoronavirus.* In this study, we proved that PABPC4 inhibited PEDV replication by degrading N protein. To test whether PABPC4 was involved in the regulation of CoV replication via N protein degradation, we cloned seven *N* genes of CoVs, including *Alphacoronavirus* (HCoV 229E), *Betacoronavirus* (SARS-CoV-2, SARS-CoV, Middle East respiratory syndrome [MERS]-CoV, and murine hepatitis virus [MHV]), *Gammacoronavirus* (IBV), and *Deltacoronavirus* (PDCoV) into the mammalian expression vector pCAGGS containing the hemagglutinin (HA) tag. These *N*-expressing plasmids were cotransfected with a plasmid encoding *FLAG-PABPC4* into HEK 293T cells for 24 h; this was then followed by co-IP. The results showed that PABPC4 protein directly interacted with each of the seven CoV N proteins (Fig. 5A). Co-IP revealed that the N proteins of different CoVs interacted with the FLAG-tagged PABPC4.

To analyze whether PABPC4 was involved in regulating the stability of N proteins, the plasmids encoding HA-tagged N proteins derived from different CoVs were cotransfected with FLAG-PABPC4 or vector control, and the N protein abundances were examined by Western blotting. The results showed that PABPC4 also downregulated the expression of the other six N proteins (Fig. 5B), and the levels of SARS-CoV-2 N protein decreased in a dose-dependent manner in response to PABPC4. These data demonstrated that PABPC4 is involved in regulating the levels of CoV N proteins.

Cells were transfected with PABPC4-FLAG for 24 h and infected with *Betacoronavirus* (MHV), *Gammacoronavirus* (IBV), or *Deltacoronavirus* (PDCoV) to investigate whether PABPC4 regulates the levels of N protein during viral infection. N2a cells were infected with MHV, H1299 cells were infected with IBV, and ST cells were infected with PDCoV. The cells and supernatants of the infected cell cultures were collected at various time points and assayed for levels of viral mRNA and N protein, as well as viral titers. As shown in Fig. 5C, G, and K, the levels of CoV N protein were significantly lower in cells overexpressing PABPC4-FLAG than in cells transfected with the control vector. CoV mRNAs in the supernatants of the cells overexpressing PABPC4 were also significantly lower than those in the cells transfected with empty vectors (Fig. 5D, H, and L). To confirm these results, we selected siRNAs targeting the different regions of PABPC4 (Fig. 5E, I, and M). Cells were transfected with PABPC4 siRNA and infected with MHV, IBV, or PDCoV at an MOI of 0.01, and the viral yield was measured by quantitative PCR (qPCR). As expected, the viral titers were significantly higher in cells transfected with the PABPC4 siRNA (Fig. 5F, J, and N). Those results suggest that PABPC4 downregulates the protein abundance of the CoV N proteins and inhibits MHV (*Betacoronavirus*), IBV (*Gammacoronavirus*), and PDCoV (*Deltacoronavirus*) replication.

### PABPC4 degrades coronavirus N proteins by selective autophagy.

**FIG 6:**
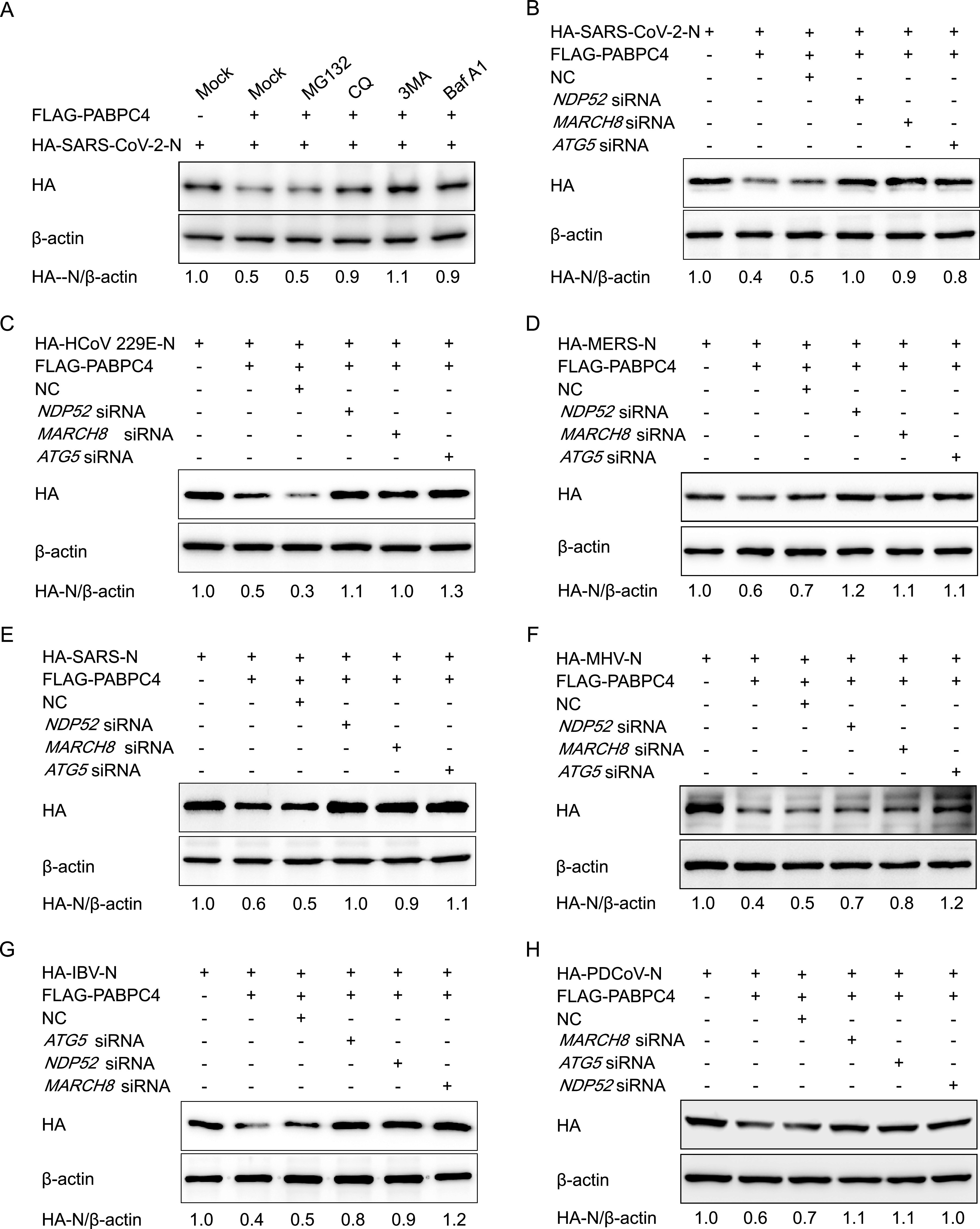
PABPC4 degrades the coronavirus N proteins through selective autophagy. (A) Plasmids encoding FLAG-PABPC4 and HA-SARS-CoV-2-N were transfected into 293T cells and then treated cells with MG132 (5 μM), 3MA (0.5 mM), CQ (10 μM), or Baf A1 (0.1 μM) for 8 h. The cell lysates were then analyzed by Western blotting. (B to H) HEK 293T cells were cotransfected with small interfering RNA (*NDP52* siRNA, *MARCH8* siRNA, *ATG5* siRNA, or negative-control siRNA) and plasmids encoding FLAG-PABPC4 and HA-SARS-CoV-2-N (B), HA-HCoV 229E-N (C), HA-MERS-N (D), HA-SARS-N (E), HA-MHV-N (F), HA-IBV-N (G), or HA-PDCoV-N (H) and then analyzed by Western blotting with anti-HA antibody. β-actin was used as the loading control.

PABPC4 degrades PEDV N protein through selective autophagy and decreases the N proteins of other *Coronaviridae* genera. So, we speculated that the PABPC4 inhibited the coronavirus replication by degrading N protein with the PABPC4-MARCH8-NDP52 autophagosome pathway. Plasmids encoding the N protein of SARS-CoV-2 were cotransfected into HEK 293T cells with PABPC4 to identify the system that predominantly mediates the degradation of coronavirus N proteins by PABPC4, and the cells were treated with the proteasome inhibitor MG132 or the autophagy inhibitor 3MA, CQ, or Baf A1. Western blotting showed that PABPC4-mediated degradation of SARS-CoV-2 N protein was blocked by the autophagy inhibitors 3MA, CQ, and Baf A1 but not by the proteasome inhibitor MG132 (Fig. 6A), which is consistent as PABPC4 degrades the PEDV N protein. To determine whether MARCH8 and NDP52 were involved in PABPC4-induced autophagic degradation of the N protein, we individually transfected HEK 293T cells with plasmids encoding HA-tagged N proteins of CoVs (SARS-CoV-2, HCOV 229E, MERS-CoV, SARS-CoV, MHV, IBV, and PDCoV) and FLAG-PABPC4, together with small interfering RNAs (*NDP52*, *MARCH8*, *ATG5*, or negative-control siRNA). At 24 h posttransfection, Western blotting was performed. We found that interfering with the expression of NDP52, MARCH8, or ATG5 effectively prevented the degradation of N proteins (Fig. 6B to H). These results showed that the PABPC4-MARCH8-NDP52 autophagosome pathway was broadly involved in CoV N protein degradation.

## DISCUSSION

**FIG 7:**
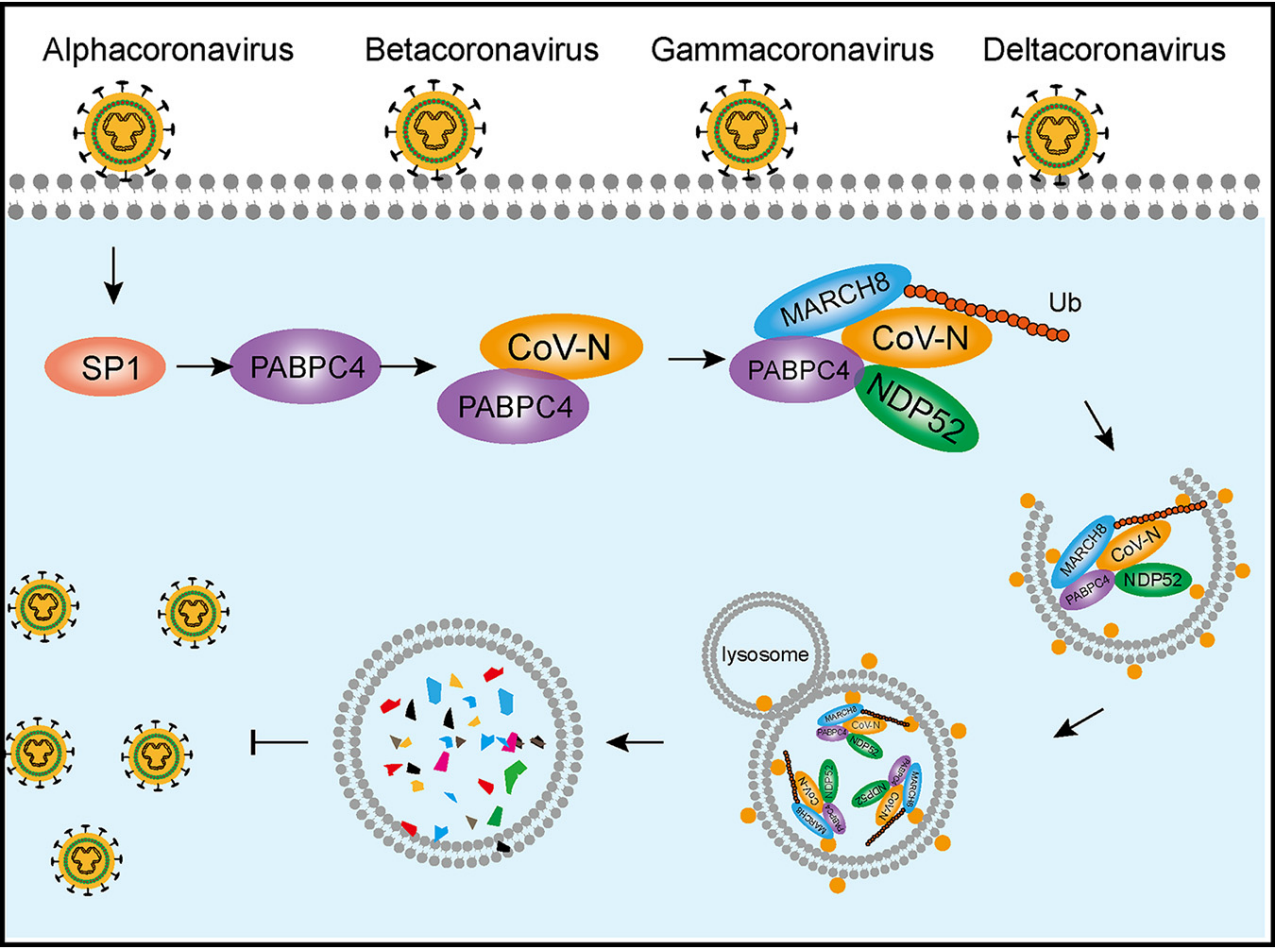
A proposed working model to illustrate the PABPC4-MARCH8-NDP52 axis broadly inhibits coronavirus replication. During coronavirus infection, transcription factor SP1 regulated host protein PABPC4, which recruited the E3 ubiquitin ligase MARCH8 to the N protein for ubiquitination. Ubiquitinated N protein was recognized by the cargo receptor NDP52, which delivered it to the autolysosomes for degradation, resulting in suppressed viral proliferation.

Coronaviruses are broadly distributed among humans, pigs, birds, and other mammals and can cause respiratory and enteric diseases (1). In the last 2 decades, coronaviruses have caused three large-scale pandemics, SARS, MERS, and SARS-CoV-2. Due to its strong transmission ability and high mortality rate, these three coronaviruses have spread rapidly worldwide, causing thousands of deaths (53). Emerging coronaviruses can cause severe diseases in humans and animals, but none of the currently available drugs or vaccines can effectively control these diseases. Therefore, the identification of effective antiviral agents to combat novel viruses is a matter of urgency. Host antiviral proteins play an important role in inhibiting viral proliferation. In this study, we revealed a novel functional property of PABPC4 in response to coronavirus infections. We found that the transcription factor of SP1 downregulated the expression of PABPC4 during PEDV infection. Our results further demonstrated that PABPC4 suppresses PEDV (*Alphacoronavirus*), MHV (*Betacoronavirus*), IBV (*Gammacoronavirus*), and PDCoV (*Deltacoronavirus*) replication by degrading the viral N protein through the PABPC4-MARCH8-NDP52 autophagosome pathway (Fig. 7) and speculated that PABPC4 inhibits coronavirus replication by targeting the viral N protein for NDP52-mediated selective autophagic degradation.

PABPC4 binds to the poly(A) tail of most eukaryotic mRNAs. It is expressed as a T-cell activation-induced protein and is also known as activated platelet protein 1 (50). PABPC4 plays an important role in the expression of erythroid mRNAs, erythroid differentiation, and regulation of telomerase activity and cell growth (54, 55). However, the antiviral function of PABPC4 and the regulation mechanism of PABPC4 during virus infection remains unexplored. In our study, we found that PABPC4 was significantly downregulated by PEDV infection. Many factors regulated gene expression. To confirm the transcription factors of PABPC4, we amplified the PABPC4 promoter sequence and found that the minimal PABPC4 core promoter was located between −153 and −1. Furthermore, we analyzed the regulatory elements of PABPC4, and the results indicated that SP1 plays a direct role in upregulating *PABPC4* expression by binding to the *PABPC4* promoter. SP1 is a zinc finger transcription factor, upregulated in response to transforming growth factor β (TGF-β) and zinc (56, 57). Upregulation of SP1 protein could serve as a useful strategy for inhibiting CoVs by promoting PABPC4 expression.

PABPC4 is one of the isoforms of cytoplasmic poly(A)-binding protein (PABP). PABP is an RNA-processing protein, which plays an important role in promoting gene expression by enhancing translation and mRNA stability (36, 37, 41). PABPC1, the best-characterized cytoplasmic PABP, could bind to the polyadenylated [poly(A)] tail of mRNAs and interacts with eukaryotic initiation factor 4G (eIF4G), which associates with the 5′ cap structure, which formed an mRNA “closed loop,” protecting mRNAs from degradation and promoting translation initiation by enhancing ribosome subunit recruitment (39, 41). PABPC4 and PABPC1 belong to the PABP family and share 75% identity at the protein level (39, 58). PABP4 is highly similar to PABP1, so PABPC4 and PABPC1 might have similar functions in regulating mRNA translation and stability. It is suggested that PABPC4 may promote viral replication, but in our study, we found that PABPC4 could inhibit the replication of PEDV, and the downregulation of the expression of PABPC4 renders cells more susceptible to PEDV replication. Furthermore, PABPC4 has been shown to combat the replication of coronaviruses. So, there should be other antiviral functions of PABPC4 to inhibit replicating a wide variety of coronaviruses. It was noted that the PEDV N protein plays an important role in virus replication and immune evasion and could be degraded by the restriction factors BST2 through the selective autophagy pathway (17, 34). Interestingly, we found that PABPC4 targeted the N proteins of coronaviruses and degraded the N proteins by selective autophagy pathway. These data indicate that PABPC4 suppresses coronavirus replication, possibly targeting and degrading viral N protein with selective autophagy.

Autophagy is a cellular catabolic process involved in maintaining intracellular homeostasis through the degradation of cellular misfolded or long-lived proteins and damaged organelles (59). Selective autophagy captures its cargo via selective autophagy receptors to degrade the viruses and virus-derived antigens (60). It has been reported that BST2 could induce selective autophagy to degrade the PEDV N protein and reduce PEDV replication (34). Here, we demonstrated that PABPC4 could recruit E3 ubiquitin ligase MARCH8 to catalyze CoV N proteins, recognized and degraded by NDP52-dependent selective autophagy. These results indicated that the induction of autophagy is beneficial for host cell antiviral factor-mediated viral restriction mechanisms. During the current coronavirus disease-19 (COVID-19; caused by SARS-CoV-2) pandemic, CQ has been administered as a first-line drug to treat this disease in several studies (61, 62), but the treatment efforts are controversial. Some studies showed that CQ did not prevent illness compatible with COVID-19 (63, 64). Chloroquine could inhibit the glycosylation of the ACE2 receptor, which acts as an entry receptor for SARS-CoV-2 (65). As an autolysosome inhibitor, it could also interfere with lysosome-mediated autophagy function. However, the inhibition of autophagy results in damaged mitochondria not being cleared via mitophagy and, thus, accumulating in cells, leading to oxidative stress and renal tubular dysfunction (66). Inhibition of autophagy pathways also inhibits the antiviral function of antiviral factors, suppressing viral replication by autophagy, such as BST2 and PABPC4. So, clinicians should carefully weigh the risks and benefits of CQ as a treatment for COVID-19.

Considering the rapid global spread of SARS-CoV-2, the development of novel strategies for the treatment or prevention of SARS-CoV-2 and other CoVs is urgent. The broad inhibition of CoV replication by the host protein, PABPC4, through the autophagy pathway provides novel targets for anticoronavirus therapeutics. Upregulating the expression of the transcription factor SP1 might increase PABPC4 expression and inhibit CoV replication. Furthermore, pharmacological modulation of the PABPC4-MARCH8-NDP52 autophagosome pathway might serve as a practical strategy for treating CoV infections, especially for COVID-19. Overexpression of PABPC4 using mammalian expression vectors or viral expression systems might be developed as plausible gene therapy for the treatment of CoV infection, especially for coronaviral diseases in animals.

## MATERIALS AND METHODS

### Cell culture and viruses.

Vero cells (African green monkey kidney cells; ATCC) and human embryonic kidney 293T cells (ATCC) were cultured in Dulbecco's modified Eagle's medium (DMEM) (Invitrogen). N2a cells and H1299 were cultured in RPMI 1640 medium supplemented with 10% fetal bovine serum (FBS) (Gibco). LLC-PK1 cells (porcine kidney cells) were kindly provided by Rui Luo (Huazhong Agricultural University) and maintained in modified Eagle's medium (MEM) (Invitrogen). All cells were incubated at 37°C with 5% CO_2_. Porcine epidemic diarrhea virus (PEDV) (JS-2013 strain) was stored in our laboratory (34, 51). Murine hepatitis virus (MHV) (strain A59 B12) was kindly provided by Susan Weiss (University of Pennsylvania). The Beaudette strain of the avian infectious bronchitis virus (IBV) (ATCC VR-22) adapted to survive in Vero cells was used in this study. PDCoV was kindly provided by Yaowei Huang (Zhejiang University).

### Antibodies and reagents.

The monoclonal antibody (MAb) against the N protein of PEDV (PEDV JS-2013) was generated using a procedure mentioned in our previous report (67). The MAb against the N protein of MHV was kindly provided by Zhaohui Qian (Chinese Academy of Medical Sciences and Peking Union Medical College). The MAb against the N protein of IBV was kindly provided by Ying Liao (Shanghai Veterinary Research Institute, Chinese Academy of Agricultural Sciences, Shanghai, China). The MAb against the M protein of PDCoV was kindly provided by Yaowei Huang (Zhejiang University, Zhejiang, China). Bafilomycin A_1_ (Baf A1) (product no. 54645), anti-LC3A/B (product no. 12741), anti-glyceraldehyde-3-phosphate dehydrogenase (GAPDH) (product no. 5174), and anti-HA-tag (product no. 3724) antibodies were purchased from Cell Signaling Technology. Anti-FLAG M2 antibody (product no. F1804), anti-Myc antibody (product no. M4439), 3-methyladenine (product no. 3-MA, M9281), chloroquine phosphate (CQ) (product no. PHR1258), and MG132 (product no. M7449) were purchased from Sigma-Aldrich. Anti-β-actin (catalog no. 60008-1), anti-GST-tag (catalog no.10000-0-AP), anti-MARCH8 (catalog no. 14119-1-AP), horseradish peroxidase (HRP)-conjugated anti-mouse IgG (catalog no. SA00001-1), and HRP-conjugated anti-rabbit IgG (catalog no. SA00001-2) antibodies were purchased from the Proteintech Group.

### Plasmids, siRNA, and transfection.

Simian siRNA (against PABPC4 and SP1 genes) and porcine siRNA (against PABPC4, HIC3, Zfx, and SP1 genes) were designed by and purchased from GenePharma (Shanghai, China), together with the control siRNA. Human ATG5 siRNA (SC-41445), human NDP52 siRNA (SC-93738), and human MARCH8 siRNA (SC-90432) were purchased from Santa Cruz Biotechnology. The mammalian expression vector p3×FLAG-CMV-7.1 was purchased from Sigma-Aldrich. The pGL3-basic vector, pRL-TK luciferase reporter plasmid, and the Dual-Glo luciferase assay system were purchased from Promega. The pEGFP-N1 plasmid, pCold TF plasmid, and pCold GST plasmid were purchased from Clontech. The pCMV-N-mCherry plasmid was purchased from Beyotime Biotechnology. All recombinant plasmids were constructed by homologous recombination using the ClonExpress II one step cloning kit (Vazyme Biotech; product no. C112-02), and the sequences were confirmed by Sanger sequencing. Seven N genes of coronaviruses (HCOV 229E, GenBank accession no. AF304460; SARS-CoV-2, GenBank accession no. MN985325; MERS, GenBank accession no. MK796425; MHV, GenBank accession no. MF618252; SARS, GenBank accession no. MK062183.1; IBV, GenBank accession no. FJ904722; PDCoV, GenBank accession no. JQ065042) with HA tags were custom synthesized by Sangon Biotech (Shanghai, PR China), and the PEDV N gene was cloned from PEDV-JS2013. The constructs were transfected using Lipofectamine 3000 Reagent (Invitrogen) according to the manufacturer's instructions. All of the siRNAs were transfected using Lipofectamine RNAiMAX (Invitrogen) according to procedures recommended by the manufacturer.

### Quantitative real-time PCR.

Total RNA was extracted using an RNeasy minikit (Qiagen) or QIAamp viral RNA minikit (Qiagen) according to the manufacturer's protocol. cDNA was synthesized using a PrimeScript RT reagent kit (TaKaRa), and SYBR qPCR master mix (Vazyme) was used for quantitative real-time PCR (qRT-PCR). Gene expression was normalized to that of β-actin or GAPDH. All of the qRT-PCRs were performed on StepOnePlus real-time PCR (Applied Biosystems).

### SDS-PAGE and Western blotting.

Cells were washed with cold phosphate-buffered saline (PBS) (pH 7.4) and lysed in radioimmunoprecipitation assay (RIPA) lysis and extraction buffer (Thermo Fisher Scientific) containing protease inhibitor cocktail (Bimake) and phosphatase inhibitor cocktail (Bimake) for 30 min on ice. The proteins in the lysates were denatured using 5× SDS-PAGE loading buffer and separated by SDS-PAGE, and the proteins were then transferred onto nitrocellulose membranes (GE Healthcare). The membranes were blocked with 5% nonfat dry milk in PBST (PBS supplemented with 0.1% Tween 20) and incubated with the primary antibodies, followed by horseradish peroxidase-conjugated secondary antibodies as described previously (34). The protein bands were quantified using ImageJ (National Institutes of Health).

### Coimmunoprecipitation.

HEK 293T cells were transfected with the indicated plasmids and lysed with NP40 cell lysis buffer (Life Technologies) supplemented with protease inhibitor cocktail. The precipitated proteins were analyzed by co-IP and Western blotting as previously described (34). Briefly, lysates were centrifuged and incubated with anti-FLAG or anti-HA antibody-bound Dynabeads Protein G (Life Technologies). The precipitated proteins were washed with 0.02% PBST and eluted from beads with elution buffer (50 mM glycine, pH 2.8) for Western blotting.

### Luciferase reporter assay.

HEK 293T cells were seeded into 24-well plates and transfected with plasmids (including Luciferase reporter plasmid and pRL-TK plasmid) using Lipofectamine 3000 (Invitrogen). Twenty-four hours later, cells were collected, and luciferase activity was measured using a Dual-Luciferase reporter assay system (Promega). Luciferase values were calculated by normalization of the firefly luciferase activity to *Renilla* luciferase activity.

### GST pulldown.

PEDV N was cloned into pCold GST plasmid (Clontech), and PABPC4 was cloned into pCold TF plasmid (Clontech). Recombinant proteins were expressed in BL21 cells (Vazyme), following which cells were lysed using the lysis buffer provided in the GST protein interaction pulldown kit (Thermo) and incubated on ice for 30 min. After centrifugation, equal amounts of GST and GST-N supernatants were mixed with PABPC4. GST pulldown assays were performed using the GST protein interaction pulldown kit according to the manufacturer's protocol. The proteins were eluted with reduced glutathione and analyzed by Western blotting.

### Immunofluorescence.

Cells were cultured on coverslips and transfected with specific expression plasmids, then fixed and permeabilized with 4% paraformaldehyde (Sigma-Aldrich) for 15 min, and then washed three times with PBS followed by 0.1% Triton X-100 (Sigma-Aldrich) for 10 min. After washing, the cells were blocked using 5% bovine serum albumin for 1 h at 37°C and then incubated with primary antibody (anti-Myc) diluted in PBS containing 5% BSA for 1 h at 37°C. The cells were then washed three times and incubated with a fluorescently labeled secondary antibody (Alexa-Fluor 568-conjugated donkey anti-mouse IgG antibody; Thermo Fisher Scientific; catalog no.A10037) for 1 h at 37°C in the dark. After the cells were washed three times with PBS, they were stained with 4′,6-diamidino-2-phenylindole (DAPI) (Beyotime Biotechnology; catalog no. C1002) at room temperature for 5 min. The cells were observed under a confocal immunofluorescence microscope (Carl Zeiss, Oberkochen, Germany).

### Statistical analysis.

All results are representative of three independent experiments. Statistical analysis was performed using GraphPad Prism 5 software. All data are expressed as mean ± standard deviation (SD). *P* values were calculated using a two-tailed Student’s *t* test or one-way analysis of variance (ANOVA).

## References

[1] Weiss SR, Leibowitz JL. 2011. Coronavirus pathogenesis. Adv Virus Res 81:85–164. doi:10.1016/B978-0-12-385885-6.00009-2. 22094080 PMC7149603

[2] Cong Y, Ren X. 2014. Coronavirus entry and release in polarized epithelial cells: a review. Rev Med Virol 24:308–315. doi:10.1002/rmv.1792. 24737708 PMC7169134

[3] Bradburne AF, Bynoe ML, Tyrrell DA. 1967. Effects of a “new” human respiratory virus in volunteers. Br Med J 3:767–769. doi:10.1136/bmj.3.5568.767. 6043624 PMC1843247

[4] Jin Y, Yang H, Ji W, Wu W, Chen S, Zhang W, Duan G. 2020. Virology, epidemiology, pathogenesis, and control of COVID-19. Viruses 12:372. doi:10.3390/v12040372. PMC7232198 32230900

[5] Fehr AR, Perlman S. 2015. Coronaviruses: an overview of their replication and pathogenesis. Methods Mol Biol 1282:1–23. doi:10.1007/978-1-4939-2438-7_1. 25720466 PMC4369385

[6] Guan Y, Zheng BJ, He YQ, Liu XL, Zhuang ZX, Cheung CL, Luo SW, Li PH, Zhang LJ, Guan YJ, Butt KM, Wong KL, Chan KW, Lim W, Shortridge KF, Yuen KY, Peiris JS, Poon LL. 2003. Isolation and characterization of viruses related to the SARS coronavirus from animals in southern China. Science 302:276–278. doi:10.1126/science.1087139. 12958366

[7] Zaki AM, van Boheemen S, Bestebroer TM, Osterhaus AD, Fouchier RA. 2012. Isolation of a novel coronavirus from a man with pneumonia in Saudi Arabia. N Engl J Med 367:1814–1820. doi:10.1056/NEJMoa1211721. 23075143

[8] Zhu N, Zhang D, Wang W, Li X, Yang B, Song J, Zhao X, Huang B, Shi W, Lu R, Niu P, Zhan F, Ma X, Wang D, Xu W, Wu G, Gao GF, Tan W, China Novel Coronavirus Investigating and Research Team. 2020. A novel coronavirus from patients with pneumonia in China, 2019. N Engl J Med 382:727–733. doi:10.1056/NEJMoa2001017. 31978945 PMC7092803

[9] Wu F, Zhao S, Yu B, Chen YM, Wang W, Song ZG, Hu Y, Tao ZW, Tian JH, Pei YY, Yuan ML, Zhang YL, Dai FH, Liu Y, Wang QM, Zheng JJ, Xu L, Holmes EC, Zhang YZ. 2020. A new coronavirus associated with human respiratory disease in China. Nature 579:265–269. doi:10.1038/s41586-020-2008-3. 32015508 PMC7094943

[10] Lin SY, Chen HW. 2017. Infectious bronchitis virus variants: molecular analysis and pathogenicity investigation. Int J Mol Sci 18:2030. doi:10.3390/ijms18102030. PMC5666712 28937583

[11] Stevenson GW, Hoang H, Schwartz KJ, Burrough ER, Sun D, Madson D, Cooper VL, Pillatzki A, Gauger P, Schmitt BJ, Koster LG, Killian ML, Yoon KJ. 2013. Emergence of porcine epidemic diarrhea virus in the United States: clinical signs, lesions, and viral genomic sequences. J Vet Diagn Invest 25:649–654. doi:10.1177/1040638713501675. 23963154

[12] Ma Y, Zhang Y, Liang X, Lou F, Oglesbee M, Krakowka S, Li J. 2015. Origin, evolution, and virulence of porcine deltacoronaviruses in the United States. mBio 6:e00064-15. doi:10.1128/mBio.00064-15. 25759498 PMC4453528

[13] Cruz JL, Sola I, Becares M, Alberca B, Plana J, Enjuanes L, Zuniga S. 2011. Coronavirus gene 7 counteracts host defenses and modulates virus virulence. PLoS Pathog 7:e1002090. doi:10.1371/journal.ppat.1002090. 21695242 PMC3111541

[14] Niederwerder MC, Hesse RA. 2018. Swine enteric coronavirus disease: a review of 4 years with porcine epidemic diarrhoea virus and porcine deltacoronavirus in the United States and Canada. Transbound Emerg Dis 65:660–675. doi:10.1111/tbed.12823. 29392870 PMC7169865

[15] Zhou P, Fan H, Lan T, Yang XL, Shi WF, Zhang W, Zhu Y, Zhang YW, Xie QM, Mani S, Zheng XS, Li B, Li JM, Guo H, Pei GQ, An XP, Chen JW, Zhou L, Mai KJ, Wu ZX, Li D, Anderson DE, Zhang LB, Li SY, Mi ZQ, He TT, Cong F, Guo PJ, Huang R, Luo Y, Liu XL, Chen J, Huang Y, Sun Q, Zhang XL, Wang YY, Xing SZ, Chen YS, Sun Y, Li J, Daszak P, Wang LF, Shi ZL, Tong YG, Ma JY. 2018. Fatal swine acute diarrhoea syndrome caused by an HKU2-related coronavirus of bat origin. Nature 556:255–258. doi:10.1038/s41586-018-0010-9. 29618817 PMC7094983

[16] Bosch BJ, van der Zee R, de Haan CA, Rottier PJ. 2003. The coronavirus spike protein is a class I virus fusion protein: structural and functional characterization of the fusion core complex. J Virol 77:8801–8811. doi:10.1128/jvi.77.16.8801-8811.2003. 12885899 PMC167208

[17] Zhang Q, Yoo D. 2016. Immune evasion of porcine enteric coronaviruses and viral modulation of antiviral innate signaling. Virus Res 226:128–141. doi:10.1016/j.virusres.2016.05.015. 27212682 PMC7111337

[18] McBride R, van Zyl M, Fielding BC. 2014. The coronavirus nucleocapsid is a multifunctional protein. Viruses 6:2991–3018. doi:10.3390/v6082991. 25105276 PMC4147684

[19] Hogue BG. 1995. Bovine coronavirus nucleocapsid protein processing and assembly. Adv Exp Med Biol 380:259–263. doi:10.1007/978-1-4615-1899-0_41. 8830489

[20] Schelle B, Karl N, Ludewig B, Siddell SG, Thiel V. 2006. Nucleocapsid protein expression facilitates coronavirus replication. Adv Exp Med Biol 581:43–48. doi:10.1007/978-0-387-33012-9_6. 17037502 PMC7123256

[21] Wu CH, Chen PJ, Yeh SH. 2014. Nucleocapsid phosphorylation and RNA helicase DDX1 recruitment enables coronavirus transition from discontinuous to continuous transcription. Cell Host Microbe 16:462–472. doi:10.1016/j.chom.2014.09.009. 25299332 PMC7104987

[22] Cong Y, Ulasli M, Schepers H, Mauthe M, V'Kovski P, Kriegenburg F, Thiel V, de Haan CAM, Reggiori F. 2020. Nucleocapsid protein recruitment to replication-transcription complexes plays a crucial role in coronaviral life cycle. J Virol 94:e01925-19. doi:10.1128/JVI.01925-19. 31776274 PMC6997762

[23] Chang CK, Lo SC, Wang YS, Hou MH. 2016. Recent insights into the development of therapeutics against coronavirus diseases by targeting N protein. Drug Discov Today 21:562–572. doi:10.1016/j.drudis.2015.11.015. 26691874 PMC7108309

[24] Lin SY, Liu CL, Chang YM, Zhao J, Perlman S, Hou MH. 2014. Structural basis for the identification of the N-terminal domain of coronavirus nucleocapsid protein as an antiviral target. J Med Chem 57:2247–2257. doi:10.1021/jm500089r. 24564608 PMC3983370

[25] Boya P, Reggiori F, Codogno P. 2013. Emerging regulation and functions of autophagy. Nat Cell Biol 15:713–720. doi:10.1038/ncb2788. 23817233 PMC7097732

[26] Kroemer G, Marino G, Levine B. 2010. Autophagy and the integrated stress response. Mol Cell 40:280–293. doi:10.1016/j.molcel.2010.09.023. 20965422 PMC3127250

[27] Kraft C, Peter M, Hofmann K. 2010. Selective autophagy: ubiquitin-mediated recognition and beyond. Nat Cell Biol 12:836–841. doi:10.1038/ncb0910-836. 20811356

[28] Into T, Inomata M, Takayama E, Takigawa T. 2012. Autophagy in regulation of Toll-like receptor signaling. Cell Signal 24:1150–1162. doi:10.1016/j.cellsig.2012.01.020. 22333395

[29] Shintani T, Klionsky DJ. 2004. Autophagy in health and disease: a double-edged sword. Science 306:990–995. doi:10.1126/science.1099993. 15528435 PMC1705980

[30] Talloczy Z, Virgin H, Levine B. 2006. PKR-dependent xenophagic degradation of herpes simplex virus type 1. Autophagy 2:24–29. doi:10.4161/auto.2176. 16874088

[31] Sun P, Zhang S, Qin X, Chang X, Cui X, Li H, Zhang S, Gao H, Wang P, Zhang Z, Luo J, Li Z. 2018. Foot-and-mouth disease virus capsid protein VP2 activates the cellular EIF2S1-ATF4 pathway and induces autophagy via HSPB1. Autophagy 14:336–346. doi:10.1080/15548627.2017.1405187. 29166823 PMC5902195

[32] Berryman S, Brooks E, Burman A, Hawes P, Roberts R, Netherton C, Monaghan P, Whelband M, Cottam E, Elazar Z, Jackson T, Wileman T. 2012. Foot-and-mouth disease virus induces autophagosomes during cell entry via a class III phosphatidylinositol 3-kinase-independent pathway. J Virol 86:12940–12953. doi:10.1128/JVI.00846-12. 22993157 PMC3497631

[33] Prentice E, Jerome WG, Yoshimori T, Mizushima N, Denison MR. 2004. Coronavirus replication complex formation utilizes components of cellular autophagy. J Biol Chem 279:10136–10141. doi:10.1074/jbc.M306124200. 14699140 PMC7957857

[34] Kong N, Shan T, Wang H, Jiao Y, Zuo Y, Li L, Tong W, Yu L, Jiang Y, Zhou Y, Li G, Gao F, Yu H, Zheng H, Tong G. 2020. BST2 suppresses porcine epidemic diarrhea virus replication by targeting and degrading virus nucleocapsid protein with selective autophagy. Autophagy 16:1737–1752. doi:10.1080/15548627.2019.1707487. 31868081 PMC8386623

[35] Wang H, Kong N, Jiao Y, Dong S, Sun D, Chen X, Zheng H, Tong W, Yu H, Yu L, Zhang W, Tong G, Shan T. 2021. EGR1 suppresses porcine epidemic diarrhea virus replication by regulating IRAV to degrade viral nucleocapsid protein. J Virol 95:e00645-21. doi:10.1128/JVI.00645-21. PMC8428380 34287043

[36] Kuhn U, Wahle E. 2004. Structure and function of poly(A) binding proteins. Biochim Biophys Acta 1678:67–84. doi:10.1016/j.bbaexp.2004.03.008. 15157733

[37] Kumar GR, Shum L, Glaunsinger BA. 2011. Importin alpha-mediated nuclear import of cytoplasmic poly(A) binding protein occurs as a direct consequence of cytoplasmic mRNA depletion. Mol Cell Biol 31:3113–3125. doi:10.1128/MCB.05402-11. 21646427 PMC3147611

[38] Gorgoni B, Gray NK. 2004. The roles of cytoplasmic poly(A)-binding proteins in regulating gene expression: a developmental perspective. Brief Funct Genomics 3:125–141. doi:10.1093/bfgp/3.2.125. 15355595

[39] Burgess HM, Richardson WA, Anderson RC, Salaun C, Graham SV, Gray NK. 2011. Nuclear relocalisation of cytoplasmic poly(A)-binding proteins PABP1 and PABP4 in response to UV irradiation reveals mRNA-dependent export of metazoan PABPs. J Cell Sci 124:3344–3355. doi:10.1242/jcs.087692. 21940797 PMC3178455

[40] Craig AW, Haghighat A, Yu AT, Sonenberg N. 1998. Interaction of polyadenylate-binding protein with the eIF4G homologue PAIP enhances translation. Nature 392:520–523. doi:10.1038/33198. 9548260

[41] Mangus DA, Evans MC, Jacobson A. 2003. Poly(A)-binding proteins: multifunctional scaffolds for the post-transcriptional control of gene expression. Genome Biol 4:223. doi:10.1186/gb-2003-4-7-223. 12844354 PMC193625

[42] Fabian MR, Mathonnet G, Sundermeier T, Mathys H, Zipprich JT, Svitkin YV, Rivas F, Jinek M, Wohlschlegel J, Doudna JA, Chen CY, Shyu AB, Yates JR, III, Hannon GJ, Filipowicz W, Duchaine TF, Sonenberg N. 2009. Mammalian miRNA RISC recruits CAF1 and PABP to affect PABP-dependent deadenylation. Mol Cell 35:868–880. doi:10.1016/j.molcel.2009.08.004. 19716330 PMC2803087

[43] Ma S, Bhattacharjee RB, Bag J. 2009. Expression of poly(A)-binding protein is upregulated during recovery from heat shock in HeLa cells. FEBS J 276:552–570. doi:10.1111/j.1742-4658.2008.06803.x. 19087191

[44] Afonina E, Stauber R, Pavlakis GN. 1998. The human poly(A)-binding protein 1 shuttles between the nucleus and the cytoplasm. J Biol Chem 273:13015–13021. doi:10.1074/jbc.273.21.13015. 9582337

[45] Harb M, Becker MM, Vitour D, Baron CH, Vende P, Brown SC, Bolte S, Arold ST, Poncet D. 2008. Nuclear localization of cytoplasmic poly(A)-binding protein upon rotavirus infection involves the interaction of NSP3 with eIF4G and RoXaN. J Virol 82:11283–11293. doi:10.1128/JVI.00872-08. 18799579 PMC2573281

[46] Salaun C, MacDonald AI, Larralde O, Howard L, Lochtie K, Burgess HM, Brook M, Malik P, Gray NK, Graham SV. 2010. Poly(A)-binding protein 1 partially relocalizes to the nucleus during herpes simplex virus type 1 infection in an ICP27-independent manner and does not inhibit virus replication. J Virol 84:8539–8548. doi:10.1128/JVI.00668-10. 20573819 PMC2919032

[47] Tsai TL, Lin CH, Lin CN, Lo CY, Wu HY. 2018. Interplay between the poly(A) tail, poly(A)-binding protein, and coronavirus nucleocapsid protein regulates gene expression of coronavirus and the host cell. J Virol 92:e01162-18. doi:10.1128/JVI.01162-18. 30209168 PMC6232474

[48] Spagnolo JF, Hogue BG. 2000. Host protein interactions with the 3' end of bovine coronavirus RNA and the requirement of the poly(A) tail for coronavirus defective genome replication. J Virol 74:5053–5065. doi:10.1128/jvi.74.11.5053-5065.2000. 10799579 PMC110857

[49] Gordon DE, Jang GM, Bouhaddou M, Xu J, Obernier K, White KM, O'Meara MJ, Rezelj VV, Guo JZ, Swaney DL, Tummino TA, Hüttenhain R, Kaake RM, Richards AL, Tutuncuoglu B, Foussard H, Batra J, Haas K, Modak M, Kim M, Haas P, Polacco BJ, Braberg H, Fabius JM, Eckhardt M, Soucheray M, Bennett MJ, Cakir M, McGregor MJ, Li Q, Meyer B, Roesch F, Vallet T, Mac Kain A, Miorin L, Moreno E, Naing ZZC, Zhou Y, Peng S, Shi Y, Zhang Z, Shen W, Kirby IT, Melnyk JE, Chorba JS, Lou K, Dai SA, Barrio-Hernandez I, Memon D, Hernandez-Armenta C, et al.. 2020. A SARS-CoV-2 protein interaction map reveals targets for drug repurposing. Nature 583:459–468. doi:10.1038/s41586-020-2286-9. 32353859 PMC7431030

[50] Yang H, Duckett CS, Lindsten T. 1995. iPABP, an inducible poly(A)-binding protein detected in activated human T cells. Mol Cell Biol 15:6770–6776. doi:10.1128/MCB.15.12.6770. 8524242 PMC230930

[51] Kong N, Wu Y, Meng Q, Wang Z, Zuo Y, Pan X, Tong W, Zheng H, Li G, Yang S, Yu H, Zhou EM, Shan T, Tong G. 2016. Suppression of virulent porcine epidemic diarrhea virus proliferation by the PI3K/Akt/GSK-3alpha/beta pathway. PLoS One 11:e0161508. doi:10.1371/journal.pone.0161508. 27560518 PMC4999130

[52] Richner JM, Clyde K, Pezda AC, Cheng BY, Wang T, Kumar GR, Covarrubias S, Coscoy L, Glaunsinger B. 2011. Global mRNA degradation during lytic gammaherpesvirus infection contributes to establishment of viral latency. PLoS Pathog 7:e1002150. doi:10.1371/journal.ppat.1002150. 21811408 PMC3141057

[53] Wang C, Horby PW, Hayden FG, Gao GF. 2020. A novel coronavirus outbreak of global health concern. Lancet 395:470–473. doi:10.1016/S0140-6736(20)30185-9. 31986257 PMC7135038

[54] Kini HK, Kong J, Liebhaber SA. 2014. Cytoplasmic poly(A) binding protein C4 serves a critical role in erythroid differentiation. Mol Cell Biol 34:1300–1309. doi:10.1128/MCB.01683-13. 24469397 PMC3993565

[55] Katzenellenbogen RA, Vliet-Gregg P, Xu M, Galloway DA. 2010. Cytoplasmic poly(A) binding proteins regulate telomerase activity and cell growth in human papillomavirus type 16 E6-expressing keratinocytes. J Virol 84:12934–12944. doi:10.1128/JVI.01377-10. 20943973 PMC3004306

[56] Chen XH, Lu LL, Ke HP, Liu ZC, Wang HF, Wei W, Qi YF, Wang HS, Cai SH, Du J. 2017. The TGF-beta-induced up-regulation of NKG2DLs requires AKT/GSK-3beta-mediated stabilization of SP1. J Cell Mol Med 21:860–870. doi:10.1111/jcmm.13025. 28165192 PMC5387140

[57] Wu L, Zhou X, Li T, He J, Huang L, Ouyang Z, He L, Wei T, He Q. 2018. Improved Sp1 and betaine homocysteine-S-methyltransferase expression and homocysteine clearance are involved in the effects of zinc on oxidative stress in high-fat-diet-pretreated mice. Biol Trace Elem Res 184:436–441. doi:10.1007/s12011-017-1214-9. 29204947

[58] Sladic RT, Lagnado CA, Bagley CJ, Goodall GJ. 2004. Human PABP binds AU-rich RNA via RNA-binding domains 3 and 4. Eur J Biochem 271:450–457. doi:10.1046/j.1432-1033.2003.03945.x. 14717712

[59] Mizushima N, Yoshimori T, Levine B. 2010. Methods in mammalian autophagy research. Cell 140:313–326. doi:10.1016/j.cell.2010.01.028. 20144757 PMC2852113

[60] Sharma V, Verma S, Seranova E, Sarkar S, Kumar D. 2018. Selective autophagy and xenophagy in infection and disease. Front Cell Dev Biol 6:147. doi:10.3389/fcell.2018.00147. 30483501 PMC6243101

[61] Cortegiani A, Ingoglia G, Ippolito M, Giarratano A, Einav S. 2020. A systematic review on the efficacy and safety of chloroquine for the treatment of COVID-19. J Crit Care 57:279–283. doi:10.1016/j.jcrc.2020.03.005. 32173110 PMC7270792

[62] Gao J, Tian Z, Yang X. 2020. Breakthrough: chloroquine phosphate has shown apparent efficacy in treatment of COVID-19 associated pneumonia in clinical studies. Biosci Trends 14:72–73. doi:10.5582/bst.2020.01047. 32074550

[63] Boulware DR, Pullen MF, Bangdiwala AS, Pastick KA, Lofgren SM, Okafor EC, Skipper CP, Nascene AA, Nicol MR, Abassi M, Engen NW, Cheng MP, LaBar D, Lother SA, MacKenzie LJ, Drobot G, Marten N, Zarychanski R, Kelly LE, Schwartz IS, McDonald EG, Rajasingham R, Lee TC, Hullsiek KH. 2020. A randomized trial of hydroxychloroquine as postexposure prophylaxis for Covid-19. N Engl J Med 383:517–525. doi:10.1056/NEJMoa2016638. 32492293 PMC7289276

[64] Mercuro NJ, Yen CF, Shim DJ, Maher TR, McCoy CM, Zimetbaum PJ, Gold HS. 2020. Risk of QT interval prolongation associated with use of hydroxychloroquine with or without concomitant azithromycin among hospitalized patients testing positive for coronavirus disease 2019 (COVID-19). JAMA Cardiol 5:1036–1041. doi:10.1001/jamacardio.2020.1834. 32936252 PMC7195692

[65] Bonam SR, Muller S, Bayry J, Klionsky DJ. 2020. Autophagy as an emerging target for COVID-19: lessons from an old friend, chloroquine. Autophagy 16:2260–2266. doi:10.1080/15548627.2020.1779467. 32522067 PMC7755324

[66] Festa BP, Chen Z, Berquez M, Debaix H, Tokonami N, Prange JA, Hoek GV, Alessio C, Raimondi A, Nevo N, Giles RH, Devuyst O, Luciani A. 2018. Impaired autophagy bridges lysosomal storage disease and epithelial dysfunction in the kidney. Nat Commun 9:161. doi:10.1038/s41467-017-02536-7. 29323117 PMC5765140

[67] Pan X, Kong N, Shan T, Zheng H, Tong W, Yang S, Li G, Zhou E, Tong G. 2015. Monoclonal antibody to N protein of porcine epidemic diarrhea virus. Monoclon Antib Immunodiagn Immunother 34:51–54. doi:10.1089/mab.2014.0062. 25723284 PMC4350141

